# Combining MRI with PET for partial volume correction improves image-derived input functions in mice

**DOI:** 10.1186/2197-7364-1-S1-A84

**Published:** 2014-07-29

**Authors:** Eleanor Evans, Guido Buonincontri, David Izquierdo, Carmen Methner, Rob C Hawkes, Richard E Ansorge, Thomas Kreig, T Adrian Carpenter, Stephen J Sawiak

**Affiliations:** Wolfson Brain Imaging Centre, University of Cambridge, Cambridge, UK; Athinoula A Martinos Centre, Harvard University, Cambridge, MA USA; Department of Medicine, University of Cambridge, Cambridge, UK; Department of Physics, University of Cambridge, Cambridge, UK; Behavioural and Clinical Neurosciences Institute, University of Cambridge, Cambridge, UK

Kinetic modelling in PET requires the arterial input function (AIF), defined as the time-activity curve (TAC) in plasma. This measure is challenging to obtain in mice due to low blood volumes, resulting in a reliance on image-based methods for AIF derivation. We present a comparison of PET- and MR-based region-of-interest (ROI) analysis to obtain image-derived AIFs from the left ventricle (LV) of a mouse model. ROI-based partial volume correction (PVC) was performed to improve quantification.

**Figure 1:**
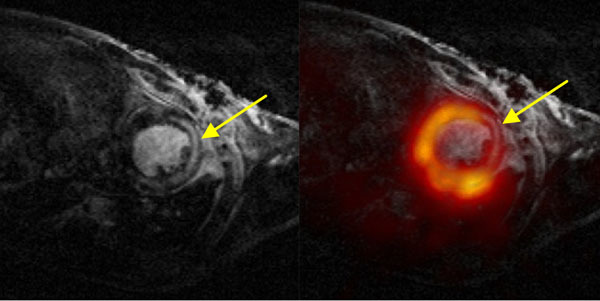
Mouse heart MR (left) and fused with ^18^F-FDG static PET (right). Arrow indicates infarcted region.

MRI and dynamic PET images were obtained from a recent study investigating treatment effects in 12 mice following myocardial infarction [1], where half the mice received a new treatment and half did not. Prospectively gated MRI (4.7T Bruker BioSpec, FLASH TR/TE 400/3ms, spatial resolution 140μm in 1mm slices) were acquired prior to PET acquisition (approx. 25MBq ^18^F-FDG bolus, 45 minute emission listmode acquisition reconstructed with 3DRP in four cardiac frames) on a split-magnet PET camera [2]. Images were co-registered using SPMMouse [3] (see Figure 1).

**Figure 2:**
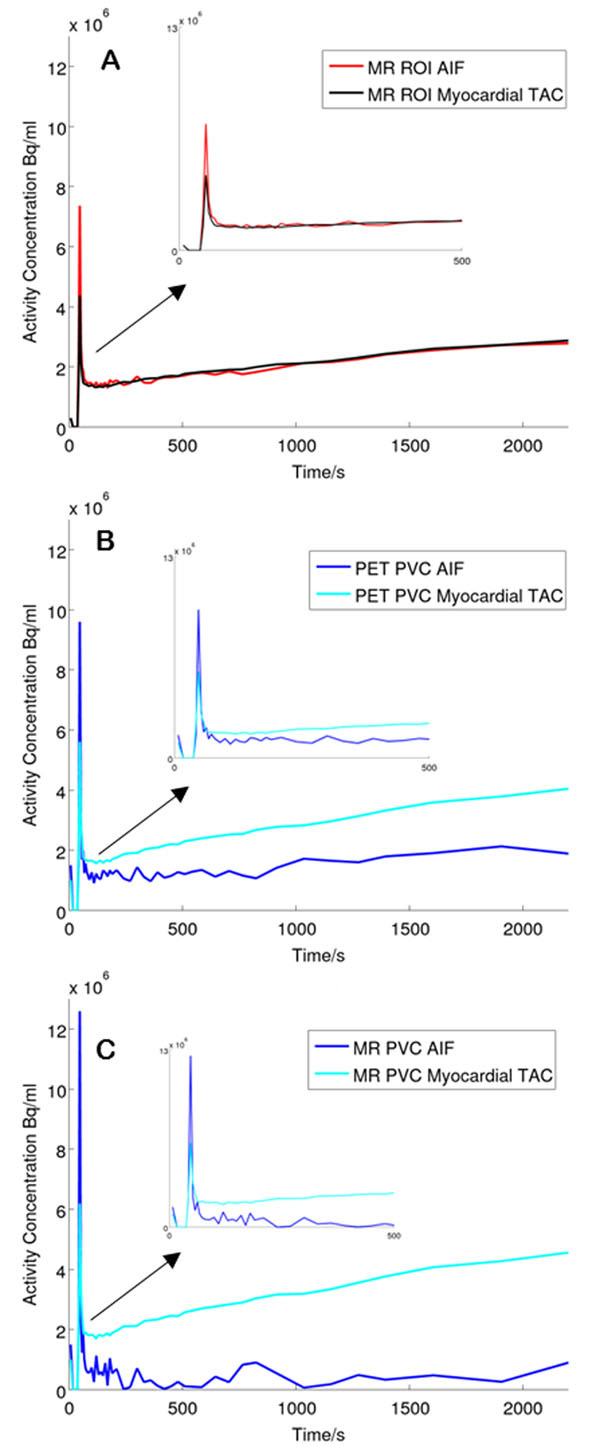
AIFs and TACs derived for single subject using (A) MR ROIs, (B) PET ROIs with PVC and (C) MR ROIs with PVC. Insets detail first 500s

*AIF extraction* AIFs were obtained by taking mean time courses from LV Lumen ROIs, shown in Figure 2. The regional geometric transfer matrix (GTM) method was applied for PVC [4], using ROIs drawn on either the co-registered MR images or directly onto the last dynamic frame PET images. ROIs covered LV lumen, myocardium, lungs/body and background. Patlak [5] analysis was performed to evaluate glucose metabolism.

Uncorrected AIFs and myocardial TACs produced by manual ROI delineation displayed contamination with myocardial signal. AIFs and myocardial curves became distinguishable if GTM PVC was applied. Only MR-based PVC produced significant differences (p<0.05) in K_i_ values between the treated and untreated groups (see Table 1).

**Table 1:** Table 1

	Glucose metabolism *K*_*i*_ (ml/min/cm^3^), Mean ± SD			
	Without PVC		With PVC	
**Group**	PET ROIs	MR ROIs	PET ROIs	MR ROIs
Untreated	0.03 ± 0.01	0.03 ± 0.01	0.4 ± 0.3	0.6 ± 0.2*
Treated	0.03 ± 0.01	0.03 ± 0.02	0.2 ± 0.3	0.2 ± 0.2*

GTM-based PVC gives best results in mice when ROIs are based on MRI data, due to its high-resolution and excellent soft-tissue contrast.
